# Ohio State Veterinary Medical Association

**Published:** 1890-09

**Authors:** W. J. Torrance

**Affiliations:** Secretary, Cleveland, O.


### SOCIETY PROCEEDINGS.

*The Ohio State Veterinary Medical Association.*—The seventh semi-annual meeting of the Ohio Veterinary Medical Association was held in the council chamber at Dayton, O., on July 16th, at 8 P. M. The President, Dr. George Butler, opened the meeting with a brief address, and said he regretted to see such a small attendance, which, however, might be partially attributed to the excessively hot weather. The minutes of the last meeting, after being read by the secretary, were amended and adopted. The secretary presented the correspondence to the meeting, and it was found that some of the members whose *addresses* had been sent to the "Chief of the Bureau of Animal Industry" had not yet received copies of the last report of said bureau. The secretary was directed to endeavor to rectify the error. A letter from Prof. Liautard, offering to publish free (or at a nominal cost, a copy for each member of our Association of the full minutes of our meetings, with the essays, etc., was discussed, and it was decided that all the American veterinary journals should be allowed an equal chance to publish the transactions of our Association.

A motion was carried accepting the resignation of Dr. Stewart, of Mt. Victory, and granting him a certificate of honorable withdrawal. Dr. W. Gribble, of Washington C. H., Ohio, now read an able paper on "Thoracic Choke," confining his remarks chiefly to the horse. He cited a large number of cases from his practical experience, and discussed the causes, symptoms and most successful modes of treatment at some length, also the varied conditions revealed to him on post-mortem examination where the cases had proven fatal, such as rupture of the œsophagus, etc. The paper proved highly interesting, and the discussion following it was participated in by almost every member present. Dr. G. W. Butler, of Circleville, O., read a paper on "Rabies and Strongylus Tetracanthus as a Coincidence in the Horse." The paper evidenced considerable scientific research on the doctor's part, and it drew forth a hearty applause and an interesting after-discussion. Dr. Shepard now read a paper entitled "A Few Practical Hints" by Dr. S. R. Howard, of Hillsboro, O. The paper was well received. The Board of Censors were abolished, and those portions of the constitution and by-laws relating thereto were amended.

Dr. G. W. Butler moved the adoption of resolutions severely censuring the Ohio Board of Live Stock Commissioners for their negligence in allowing the disposal of glandered horses in Piqua County, O., and requested that a copy of said resolutions be recorded in the minutes of our meeting, and also sent to said Board of Live Stock Commissioners and to the Governor of Ohio. After a heated discussion Dr. Butler's resolutions were carried.

A vote of thanks was tendered the city council of Dayton, O., for the use of the council chamber. The secretary was ordered to urge the members of our Association to attend the next meeting of the United States Veterinary Medical Association, at Chicago.

Meeting then adjourned. Most of the members remained over in Dayton the following day and were escorted through the Soldiers' Home by Dr. Howe, and also witnessed an operation upon a "roarer" at Dr. Shaw's Veterinary Infirmary. Dr. G. W. Butler administered the chloroform while Drs. Shaw, Wight and Gribble assisted one another in the removal of the left arytenoid cartilage and thyro-arytenoidean ligament.

W. J. TORRANCE, *Secretary*, Cleveland, O.

